# Book Review

**Published:** 1909-03

**Authors:** 


**Clinical Methods.** By Robert Hutchison, M.D., and Harry Rainy, M.D. Fourth Edition. Pp. xv., 632. London: Cassell and Company Limited. 1908.—This volume is so well known that it requires little comment. Having reached its fourth edition, the whole work has been revised and brought up to date. There are many additions and some alterations. The book is one which we think should be in the hands of every clinical clerk.

**Physical Signs of Diseases of the Thorax and Abdomen.** By James E. H. Sawyer, M.D. Pp. xi., 188. London: Baillière, Tindall & Cox. 1908.—The methods of examining the physical signs in the lungs, heart and abdomen, are carefully described in this book, and tables of differential diagnosis of several diseases are included. As the book is written for medical students, we think it a pity that the author has not made it more extensive by including all the physical signs which a student is required to note when taking a case. For example, the physical signs of the nervous and some other systems are not described. Perhaps in a second edition the utility of the book may be increased by extension in scope.

**Studies in Blood Pressure.** By George Oliver, M.D. Second Edition. Pp. xii., 255. London: H. K. Lewis. 1908.—The second edition of this little book has been considerably enlarged, and contains in a small space a very large amount of most useful clinical experience. The author, having reason to suppose from his observations that the blood pressure readings obtained by the armlet method were in many cases erroneous, has used his hæmo-dynamometer with, he believes, more accurate results.

This instrument is designed to balance the blood pressure in an artery, not to close it. Arterio-sclerotic conditions being those which more particularly interfere with a true estimation of the systolic pressure, the author recommends the use of his instrument in these states. Chapters written in a practical style are devoted both to the treatment of supernormal and subnormal arterial pressure.

**Electric Ions and their Use in Medicine.** By Stephane Leduc. Translated by R. W. Mackenna, M.A., M.B., Ch.B. Pp. vii., 70. London: Rebman Limited. 1908.—This little book gives a short summary of the theory and practice of electrolysis as applied to therapeutics. Professor Leduc, who, as the translator observes, is a pioneer in this matter, devotes three chapters out of the six into which the book is divided to a general account of the theory of electrolytic dissociation. They are marked by extreme lucidity and succinctness, and will be found especially useful to those whose recollection of these subjects is becoming somewhat hazy. The remainder of the book is devoted to the practical applications of the ionic condition of matter to medical purposes. There appears to be no doubt that considerable penetration of the tissues can be effected by making use of positive or negative ions, and the main practical point is to use suitable appliances, and to be accurate in the details of the technique. In some cases Dr. Leduc, like most enthusiasts, proves that we can be sure of eating our cake and also having it, as in the application of glycerine of carbolic acid instead of a watery solution. Surely the fact that dissociation hardly takes place at all in the former, besides rendering it a non-caustic application, also diminishes its antiseptic effect. In the treatment of certain nervous and cerebral disorders, it seems likely that Dr. Leduc's methods are no better than others which are less elaborate and costly; but for the local application of drugs through the unbroken skin, electrolytic dissociation appears to be a useful and effective condition. The book is fluently translated, and in a short appendix the translator has added a few notes on various practical therapeutic points.

**Introduction to Dermatology.** By Norman Walker, M.D., F.R.C.P. Fourth Edition. Pp. xvii., 332. Edinburgh: William Green & Sons. 1908.—The principal addition in the fourth edition of this well-known work consists of colour-reproductions of casts made for the author by Dr. Cranston Low. The value of the book as a guide in dermatology is undoubtedly increased by such representations of actual diseases, more particularly since nomenclature in skin diseases is passing through a stage of complete transition. How ready the author is to welcome a more rational nomenclature may be judged from his definition of eczema as "the term commonly applied to any wet or scaly inflammation of the skin, of the cause or nature of which the observer is ignorant." He continues: "To those who know nothing, or next to nothing, of the diseases of the skin, most eruptions are eczema. The greater the observer's experience, the more diseases can he differentiate from eczema, and the fewer remain to be so called." Of pityriasis he says, "The use of the simple term is merely a pedantic method of concealing ignorance." As regards vaccine treatment we quote his opinion in full: "If I have damned the vaccine treatment of acne with faint praise, I am ready to make amends in connection with sycosis. I have seen cases, which I have treated in vain with all the other methods, improve so strikingly under injections of vaccine prepared from cultures of their own staphylococci that, so far as concerns sycosis, I am prepared to shout with the loudest of the vaccinists." Although the book is no more than an introduction to the subject, it teaches one how to approach a skin disease with a view to diagnosis and treatment, and cannot fail also to have some influence in shaking off the yoke of the past, and freeing dermatology from its mediæval superstitions and its heritage from the leechdoms.

**Reports of the Society for the Study of Disease in Children.** Vol. VII. Session 1906–7. London: J. & A. Churchill.—The present volume fully maintains the standard of interest which has been shown by its six predecessors. The index, which is very carefully and exhaustively compiled, shows the large number of rare and interesting cases which are annually brought before the Society, especially in the department of infantile deformities and congenital abnormalities. The Wightman Lecture, given last year by Dr. Frederick Taylor, is a valuable contribution to our knowledge, and an excellent example of the exhaustive and accurate methods of its author. The relative incidence of lobar and lobular pneumonia in children is very carefully discussed, and the prognosis in each form considered in detail. A short but suggestive review of the treatment is also given. Dr. Taylor's lecture will no doubt become a *locus classicus* for future writers on this important subject. There is also an interesting discussion on Rickets by various leading authorities, and other valuable contributions include a paper on "Nephritis in Infants," by Dr. George Carpenter; one on "Cerebro-spinal Meningitis," by Dr. Edmund Cautley; and two on "Various Aspects of Rheumatism in Children," by Dr. C. O. Hawthorne.

**Our Baby.** By Mrs. J. Langton Hewer. Eleventh Edition. Pp. viii., 168. Bristol: John Wright & Sons, Ltd. 1908.—Though elementary in character, this book will be found quite comprehensive enough for all practical purposes. The chapters on diet and bottle-feeding are especially good, though a great many now advocate a higher percentage of milk for bottle-fed babies at birth than that recommended in food-table. We think the idea of describing in detail the preparation of peptonised milk, raw meat juice, albumen water, bread jelly, &c., a good one, for medical men are apt to overlook such details of cookery. The dress, food, exercise, sleep, and accidents and illnesses of children, are all described in this book minutely. It would certainly be advisable if in future editions such drugs as potassium bromide, recommended for excitable children, were eliminated from the chapter on medicines as liable to grave misuse. Altogether, we believe that this little book will nail many " old wives' fables " to the counter.

**Annual Report of the Board of Regents of the Smithsonian Institution for the Year ending June 30th, 1906.** Washington: Government Printing Office. 1907.—There are fewer papers on medical topics than usual in this year's report, the only one being on " Zoology and Medicine," by Raphael Blanchard, and a most interesting and suggestive paper it is. There are several communications on electrical subjects, including a paper by Madame Curie on " Modern Theories of Electricity and Matter." To the general reader we would recommend papers on " Iceland: its History and Inhabitants "; and two papers on the " Rôle of Chemistry in Painting," and on " Oils, Varnishes and Mediums used in the Painting of Pictures."

**Lectures on Babies.** By RALPH VINCENT, M.D., B.S. Pp. viii., 113. London: Baillière, Tindall & Cox. 1908.—The preface to this small volume informs us that its contents consist of lectures delivered at the Infants' Hospital. A perusal of it leaves us in some doubt as to the class of audience to whom they were delivered. Much of the matter is far too sketchy and elementary in character for an audience of medical men or even medical student s. For instance, the account of the pathology of rickets is far too superficial, and the notes on such matters as hypertrophic stenosis of the pylorus (so-called), the absorption of food, and the composition of milk, are almost in the style of " every mother her own doctor." On the other hand, if the lectures were delivered to an audience of nurses, surely it was very out of place to include such things as the formulæ for olein, palmatin and stearin, the terminology of the summer diarrhœa of infants, and the question as to operation for a contracted pylorus, which are either incomprehensible to those not having special scientific training or are beyond the scope of ordinary nursing work. The chapter of most interest is that in which Dr. Vincent describes the model dairy and milk laboratory of the Infants' Hospital; for although there is nothing very new in the details, as far as can be gathered from the short descriptions given, it is always satisfactory to know that these institutions can be efficiently worked in spite of the hostile criticism to which they have been subjected in some quarters. The book would have been much improved had the colloquial lecture style been modified to suit the conditions of printed matter. There are several minor inaccuracies to be noted. The author, for instance, fails to distinguish between precipitation and coagulation, between the reaction of the intestinal wall and the intestinal contents, or between pyloric spasm and hyperplasia of the fibrous and muscular tissues. We are, too, at a loss to conjecture how "atrophy" can etymologically mean "loss of function." Dr. Vincent objects to the term "epidemic" diarrhœa on the ground that babies suffering from this disease do not communicate it to other babies when in hospital. On parallel lines enteric fever might be removed from the list of epidemic diseases and placed among local intestinal affections.

**Some of the Clinical Aspects of Pneumonia.** By Donald W. C. Hood, C.V.O., M.D. Pp. viii., 117. London: John Bale, Sons & Danielsson, Ltd. 1907.—The substance of clinical lectures and demonstrations, delivered by the author at the West London Hospital to postgraduates, forms the basis of this book, which is full of ripe clinical experience and sound common sense. The author deals fully with the different clinical aspects of the disease in its very various manifestations, and in order to make his points more clear, describes actual cases which have come under his own observation. He has some interesting remarks to make in his last chapter, which deals with the principles of treatment, on the somewhat vexed question of the administration of alcohol in this disease.

**Nodal Fever (Febris nodosa).** By Alfred Austin Lendon, M.D. Pp. vii, 90. London: Baillière, Tindall & Cox. 1905.—The synonyms Erythema nodosum and Erythema multiforme are a sufficient explanation of the disease which Dr. Lendon wishes to nominate as above. He looks upon the malady as an acute specific infectious fever, rather than an affection of the skin, as has commonly been taught. An analysis of some sixty-three cases, studies for the most part in Adelaide, has convinced him that erythema nodosum is preceded and attended by pyrexia with general signs of malaria, that its course is marked by a prodromal period, by a stage of eruption, and by a period of convalescence; and that it occurs under conditions which strongly suggest infection from one case to another. He gives much evidence in favour of this view, but does not quite eliminate the rheumatic element or convince us that some other than the rheumatic germs are the cause of the disease.

